# Acute Effects of Mental Activity on Response of Serum BDNF and IGF-1 Levels in Elite and Novice Chess Players

**DOI:** 10.3390/medicina55050189

**Published:** 2019-05-22

**Authors:** Hamid Arazi, Hanieh Aliakbari, Abbas Asadi, Katsuhiko Suzuki

**Affiliations:** 1 Department of Exercise Physiology, Faculty of Sport Sciences, University of Guilan, Rasht 1438, Iran; 2 Bandar-e-Anzali Branch, Islamic Azad University, Bandar-e-Anzali 43111, Iran; aliakbari.hanieh@gmail.com; 3 Department of Physical Education and Sport Sciences, Payame Noor University, Tehran 19395-3697, Iran; Abbas_asadi1175@yahoo.com; 4 Faculty of Sport Sciences, Waseda University, Tokorozawa 359-1192, Japan; katsu.suzu@waseda.jp

**Keywords:** brain, neurotrophic factor, mental activity

## Abstract

*Background and Objectives:* Although the effects of physical exercise on brain functions are well studied, the influence of mental activity is unknown. The aim of this study was to assess the influence of a session of mental activity on brain neurobiological factors in chess players. *Materials and Methods:* Ten elite and novice chess players were recruited to participate in this study as volunteers. The subjects performed a session of standard chess matches as a mental activity. Before and after each chess match, blood samples were drawn to analyze changes in serum brain-derived neurotrophic factor (BDNF) and insulin-like growth factor 1 (IGF-1). *Results:* After each chess match, both the elite and novice groups showed significant increases in serum BDNF and IGF-1 concentrations. The elite group also showed significantly greater changes in BDNF and IGF-1 levels (*p* ≤ 0.05) than the novice group. *Conclusions:* Our findings indicate that a session of standard chess matches as a mental activity is effective for elevating BDNF and IGF-1 levels, and that their elevation in elite players seems to be more pronounced than those in novice players.

## 1. Introduction

Brain-derived neurotrophic factor (BDNF) is a member of the neurotrophins family that plays an important role in neuronal transmission, modulation, and plasticity for the development of adulthood [1,2]. In fact, BDNF plays a key regulatory role in the growth and development of neurons in many areas of the brain, and also resistance to nerve damage. Insulin-like growth factor 1 (IGF-1) is related to neurogenesis and the regulation of the *BDNF* gene [3], and is involved in the growth and differentiation of neurons [4].

It has been suggested that increases in IGF-1 are associated with increases in BDNF, resulting in increased hippocampal synaptic plasticity and the increased expression of molecules associated with learning and cognitive tasks following physical exercise [1]. On the other hand, physical exercise enhances the production of IGF-1 and also affects the brain by enhancing BDNF [1]. To date, it has been well documented that physical exercise regulates BDNF and IGF-1 expression and enhances brain plasticity [2,4,5,6]. Although there is clear evidence of physical-activity-induced increases in BDNF and IGF-1, the effect of mental activity on serum BDNF and IGF-1 levels remains unclear.

Brain exercises are a widely known approach to preventing dementia, increasing neurogenesis, and protecting neurons. The game of chess has been practiced as a brain activity for many years. Playing the game involves many aspects of cognition, improves mental health, and plays a critical role in enhancing brain cell stimulation [7,8]. In fact, mental activity has the potential to increase motivation in individuals and has been known to improve working memory and verbal episodic memory [7]. However, the influence of a standard mental activity (i.e., a chess game) on serum BDNF and IGF-1 is unclear. Some studies have reported that long-term mental activity has positive effects on BDNF and cognitive function [7,8]. To date, no studies have been conducted on the effects of a session of chess as a standard mental activity on BDNF and IGF-1 changes in elite chess players who have had at least five years of experience in this mental activity in comparison to novice players. In addition, determining the role of a session of a standard mental activity on peripheral BDNF and IGF-1 levels is unclear. Therefore, this study had three aims, including (1) monitoring the resting BDNF and IGF-1 levels of elite and novice chess players, (2) determining acute effects of a session of a standard chess game on BDNF and IGF-1 changes, and (3) determining the changes between these variables in elite and novice chess players.

## 2. Materials and Methods

### 2.1. Participants

The subjects of this study were 10 elite male chess players who had played chess for more than five years (age 31.0 ± 1.1 years, weight 82.5 ± 5.6 kg, height 180.3 ± 7.5 cm, and chess game experience 5.7 ± 6.2 years) and 10 novice male chess players who had played chess for less than one year (age 26.3 ± 2.1 years, weight 75.7 ± 6.2 kg, height 179.5 ± 5.4 cm, and chess game experience 1.4 ± 3.6 years) and participated in the chess tournament in this study as volunteers. All subjects were carefully informed about the experimental procedures and about the benefits associated with participation in the study, which was approved by the Declaration of Helsinki and Ethics Committee for the University of Guilan and Islamic Azad University (date of approval and the project identification code are respectively 21 September 2016 and IAU1395631). Thereafter, the subjects signed a written consent form before participating in the study.

### 2.2. Study Design

This study utilized a cross-sectional design to assess the role of chess experience on brain neurobiological factors after the chess match. Before participating, subjects were familiarized with the study design and testing procedures. During this session, subject characteristics such as age, height, and weight were assessed. Subjects who participated in the standard chess tournament completed a session of chess (i.e., 180 min) in the afternoon. Before and after each chess game, blood samples were withdrawn to analyze serum BDNF and IGF-1 levels.

### 2.3. Anthropometric Measurements

Height was measured to the nearest 0.5 cm using a wall-mounted stadiometer (Seca 222, Terre Haute, IN, USA). Weight was measured to the nearest 0.1 kg using a digital scale (Tanita, BC-418MA, Tokyo, Japan).

### 2.4. Mental Activity

In this study, a standard chess match was used to measure mental activity in chess players. Each subject performed a 90 min chess match and received 30 s for each movement. Overall, all subjects completed 180 min of standard chess.

### 2.5. Blood Sampling and Analysis

Blood samples were withdrawn (5 cc) from the antecubital vein into plain evacuated test tubes. The blood was allowed to clot at room temperature for 30 min and centrifuged at 3000× *g* for 15 min. The serum layer was removed and frozen at −80 °C in multiple aliquots for further analyses. All samples were assayed in duplicate and were decoded only after completing the analyses. Serum IGF-1 was measured by a commercially available enzyme-linked immunosorbent assay (ELISA) kit (Diamerta, R&D manufacturing, Via Pozzuolo, Italy) according to the manufacturer’s procedures. The sensitivity of the IGF-1 assay was 8.7 ng/mL. An intra-assay coefficient of variation (CV) for IGF-1 was less than 6%. Serum BDNF was assayed in duplicate according to the manufacturer’s instructions (R&D Systems, Inc., Minneaplis, MN, USA). The sensitivity of the BDNF assay was 20 pg/mL.

### 2.6. Statistical Analyses

All data are presented as the mean ± standard deviation (SD). Normality of all data before and after interventions was checked with the Shapiro–Wilk test. To determine the effect of interventions on neurobiological changes, a two-way analysis of variance with repeated measurements (2 [groups] × 2 [times]) was applied. Pearson product moment correlation coefficient (r) was used to determine relationship between BDNF and IGF-1 using post-test scores. Effect sizes (ES) were calculated using Cohen’s d. The magnitude of the ES statistics was considered trivial <0.20; small, 0.20–0.50; moderate, 0.50–0.80; large, 0.80–1.30; and very large >1.30. For each variable, a percent change score was calculated ((post − pre) /pre × 100). The level of significance was set at *p* ≤ 0.05.

## 3. Results

**Figure 1:**
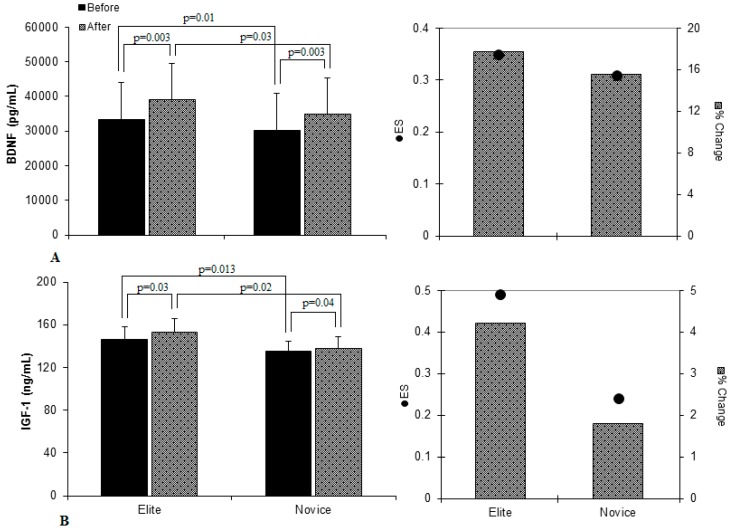
Changes in dependent variables from before to after chess match. Values are mean ± standard deviation (SD). (**A**) BDNF levels; (**B**) IGF-1 levels.

There was a significant difference between groups before chess match in resting BDNF levels (*p* = 0.01). In addition, both the elite (from 33,300 ± 10,800 to 39,200 ± 10,500 pg/mL, *p* = 0.003) and novice (from 30,200 ± 10,600 to 34,900 ± 10,400 pg/mL, *p* = 0.003) players demonstrated moderate significant increases in the BDNF levels after chess match and these increases were minimally greater for the elite chess players (ES = 0.35 vs. 0.31, % change = 17.7 vs. 15.5, respectively) (*p* = 0.03) (Figure 1A).

Similarly, there was a significant difference between groups before chess match in resting IGF-1 levels (*p* = 0.013). In addition, both the elite (from 146.6 ± 11.7 to 152.7 ± 13.3 ng/mL, *p* = 0.03) and novice (from 135.2 ± 9.5 to 137.69 ± 11.1 ng/mL, *p* = 0.048) players demonstrated moderate significant increases in the IGF-1 levels after chess match and these increases were greater for the elite chess players (ES = 0.49 vs. 0.24, % change = 4.2 vs. 1.8, respectively) (*p* = 0.02) (Figure 1B).

In addition, no statistically significant correlations were detected between serum BDNF and IGF-1 levels for the elite (r = 0.4) and novice (r = 0.19) chess players after match (*p* ≥ 0.05).

## 4. Discussion

Our findings showed that an acute session of standard chess matches induced meaningful changes in BDNF and IGF-1 and the elite chess players showed greater elevations compared to novice chess players. However, we did not find significant correlations between IGF-1 and BDNF changes after chess match for the elite and novice players. In line with our findings, previous studies [1,2,9,10] reported that physical exercise induced positive effects on the elevation of circulating BDNF and IGF-1 levels; however, the influence of mental activity on BDNF and IGF-1 is less investigated. In accordance with acute effects of mental activity on BDNF, Lin et al. [7] examined the effects of one session of Chinese GO-match on BDNF levels in Alzheimer’s disease (AD) and found that Chinese GO-game intervention ameliorates AD manifestations by upregulating BDNF levels. These findings revealed that mental activity plays an important role in BDNF secretion. The possible mechanism(s) for the elevation of serum BDNF levels could be due to increase in the mitogen-activated protein kinase (an important intracellular signal pathway involved in BDNF production and secretion) in the hippocampus and brain cortex [11,12]. On the other hand, mental activity could increase mature BDNF (mBDNF) through changing some signal pathways (i.e., Ca2+/calmodulin-dependent protein kinase II (CaMKII) and Synapsin I), increase expression and activation of tissue plasminogen activator (tPA), and therefore enhance proteolytic mRNA of mBDNF [13]. Although we did not examine the molecular pathway of BDNF secretion, here we assume that increase in IGF-1 concentration could be another explanation, since IGF-1 easily passes through the blood–brain barrier and induces the transformation of proBDNF to mBDNF in the central nervous system, resulting in serum BDNF enhancements [14,15].

Following the chess match, serum IGF-1 increased significantly compared to prematch for both the elite and novice groups. However, the magnitude of change for the elite players was greater than for novice players. To date, little is known about the influence of mental activity on IGF-1 responses; however, with regard to findings of this study, it seems that mental activity could increase IGF-1 levels which are in line with previous studies that examined the effects of physical exercise on IGF-1 levels. In fact, regarding positive effects of IGF-1 on BDNF levels [14,16], IGF-1 could be a factor contributing to neural cell growth, differentiation, and survival.

Taken together, a session of a chess match as a mental activity not only increased IGF-1 secretion but also increased serum BDNF level in chess players. This finding theoretically confirms the high potential of brain neurotrophic factors in chess players to boost neuronal plasticity and neurogenesis [15,17].

## 5. Conclusions

The present study indicates that long-term experience in chess game induces a greater resting level in serum BDNF and IGF-1 and a session of chess match induces significant elevations in BDNF and IGF-1 levels which may induce beneficial metabotropic and neurotropic effects. In clinical importance, the chess match as a standard mental activity is recommended to increase serum IGF-1 and BDNF levels and possibly may be used to prevent brain dementia.
